# Behavioral photosensitivity of multi-color-blind medaka: enhanced response under ultraviolet light in the absence of short-wavelength-sensitive opsins

**DOI:** 10.1186/s12868-023-00835-y

**Published:** 2023-12-14

**Authors:** Kiyono Mizoguchi, Mayu Sato, Rina Saito, Mayu Koshikuni, Mana Sakakibara, Ran Manabe, Yumi Harada, Tamaki Uchikawa, Satoshi Ansai, Yasuhiro Kamei, Kiyoshi Naruse, Shoji Fukamachi

**Affiliations:** 1 Laboratory of Evolutionary Genetics, Department of Chemical and Biological Sciences, Japan Women’s University, Mejirodai 2-8-1, Bunkyo-Ku, Tokyo 112-8681 Japan; 2 Laboratory of Bioresources, National Institute for Basic Biology, Aichi, 444-8585 Japan; 3 Graduate School of Life Sciences, Tohoku University, Miyagi, 980-8577 Japan; 4 Present Address: Laboratory of Genome Editing Breeding, Graduate School of Agriculture, Kyoto University, Kyoto, 606-8507 Japan; 5 Spectrography and Bioimaging Facility, National Institute for Basic Biology, Aichi, 444-8585 Japan; 6 Department of Basic Biology, School of Life Science, The Graduate University for Advanced Studies (SOKENDAI), Aichi, 444-8585 Japan

**Keywords:** Color vision, Spectral sensitivity, Ultraviolet (UV), Cone opsin, Optomotor response (OMR), Medaka

## Abstract

**Background:**

The behavioral photosensitivity of animals could be quantified via the optomotor response (OMR), for example, and the luminous efficiency function (the range of visible light) should largely rely on the repertoire and expression of light-absorbing proteins in the retina, i.e., the opsins. In fact, the OMR under red light was suppressed in medaka lacking the red (long-wavelength sensitive [LWS]) opsin.

**Results:**

We investigated the ultraviolet (UV)- or blue-light sensitivity of medaka lacking the violet (short-wavelength sensitive 1 [SWS1]) and blue (SWS2) opsins. The *sws1/sws2* double or *sws1/sws2/lws* triple mutants were as viable as the wild type. The remaining green (rhodopsin 2 [RH2]) or red opsins were not upregulated. Interestingly, the OMR of the double or triple mutants was equivalent or even increased under UV or blue light (λ = 350, 365, or 450 nm), which demonstrated that the rotating stripes (i.e., changes in luminance) could fully be recognized under UV light using RH2 alone. The OMR test using dichromatic stripes projected onto an RGB display consistently showed that the presence or absence of SWS1 and SWS2 did not affect the equiluminant conditions.

**Conclusions:**

RH2 and LWS, but not SWS1 and SWS2, should predominantly contribute to the postreceptoral processes leading to the OMR or, possibly, to luminance detection in general, as the medium-wavelength-sensitive and LWS cones, but not the SWS cones, are responsible for luminance detection in humans.

**Supplementary Information:**

The online version contains supplementary material available at 10.1186/s12868-023-00835-y.

## Background

Animals can detect a range of electromagnetic waves as visible light. This range is 380–770 nm for humans (CIE 1931 color space [1]), although waves at shorter or longer wavelengths (ultraviolet [UV] or infrared [IR], respectively) become visible under optimized conditions. For example, the authors could perceive UV (λ = 350 nm) of 15 μmol/m^2^/s and IR (λ = 820 nm) of 100 μmol/m^2^/s during previous experiments using the Okazaki Large Spectrograph [2–6].

The range of visible light perception varies among animals; e.g., many insects perceive UV light at shorter wavelengths than do humans [7]. The behavioral UV sensitivity of animals has been demonstrated by analyzing, for example, phototaxis [8], the dorsal light response [9], body tilt [10], body-color change [11], agonistic/courtship display [12], maze training [13], or the optomotor response (OMR) [9]. Photopic perception of UV light is believed to rely on the cone opsin called short-wavelength sensitive type 1 (SWS1), the absorption maximum (λ_max_) of which is shorter (360–450 nm) than those of other cone opsins, i.e., SWS type 2 (SWS2), rhodopsin type 2 (RH2), or long-wavelength sensitive (LWS) [14, 15]. However, direct evidence supporting this genotype–phenotype relationship is scarce, as exemplified below.

Mammals (with the exception of monotremes) have only SWS1 and LWS in the retina. The eyes of *SWS1*-knockout (KO) mice became electrophysiologically insensitive to UV light (λ = 360–365 nm) [16, 17]. Tritanope individuals lacking SWS1 exhibit a reduced luminous efficiency of violet/blue light [18]. These results support an exclusive role for SWS1 in the perception of UV light or light at short wavelengths. However, such evidence in fish remains more obscure. Zebrafish with the mutated *tbx2b* gene exhibit differentiation of SWS1-cone precursors into rods and the lack of dispersion of melanophores in response to dorsal illumination using near-UV light [11]. Acute chemical ablation of SWS1 cones in larval zebrafish reduced the sensitivity to blue and UV light but was quickly recovered within 48–72 h [19]. Similar acute ablation of SWS1 cones reduced foraging performance under UV light at 1 day after the ablation in zebrafish larvae [20]. An expressional switch from SWS1 to SWS2 triggered by the thyroid hormone in rainbow trout also reduced foraging performances, possibly because of the decreased UV contrast of its prey, *Daphnia* [21]; however, the *SWS1*-KO rainbow trout exhibits a malformation in the eyes and head [22].

Using medaka (*Oryzias latipes* or *Oryzias sakaizumii*), we established several types of “color-blind” strains by knocking out the *cone-opsin* genes for studying genotype–phenotype relationships in color vision in animals. The strain lacking LWS (LWSa and LWSb; λ_max_ = 561 or 562 nm) did not exhibit the OMR under red light (λ ≥ 730 nm), whereas the wild-type (WT) counterpart did, up to λ = 830 nm [2, 3]. The *lws* mutant also showed a reduced body-color preference during mate choice, possibly because of a decreased ability for color discrimination [23]. The strain lacking SWS2 (SWS2a and SWS2b; λ_max_ = 439 or 405 nm) similarly exhibited a reduced body-color preference [5]. However, the *sws2* mutant exhibited the OMR under blue light (λ = 400 or 440 nm) as sensitively as the WT counterpart [5], likely because either 1) the absence of the blue opsin was compensated by the neighboring violet and/or green opsins, or 2) the blue opsin is not associated with the OMR.

We recently established another color-blind strain lacking SWS1 (λ_max_ = 356 nm; [24]). Unlike the *SWS1*-KO rainbow trout [22], the medaka *sws1* mutant was fully viable and retained the ordinary square-mosaic distribution of cones in the retina. In this study, we first focused on its behavioral phenotypes, i.e., the body-color preference under white light and the OMR under UV light. Based on the absence of apparent differences between the WT animals and the *sws1* mutants (see the Results), we further established the *sws1/sws2*-double and *sws1/sws2/lws* triple mutants and characterized their behavioral photosensitivity based on the OMR.

## Results

### Mate choice of the *sws1* mutant

The body colors of the *color interfere* (*ci*) mutant and the Actb–SLα:GFP transgenic strain are pale gray and dark orange, respectively [25, 26]. Their genomes are identical, with the exception of the transgene in Actb–SLα:GFP, which expresses a hormone (somatolactin alpha [SLα]) and *Renilla* green fluorescent protein (GFP) ectopically. Our previous experiments repeatedly demonstrated that these color variants mate assortatively; i.e., males strongly prefer females of the same strain [27–30].

**Fig. 1:**
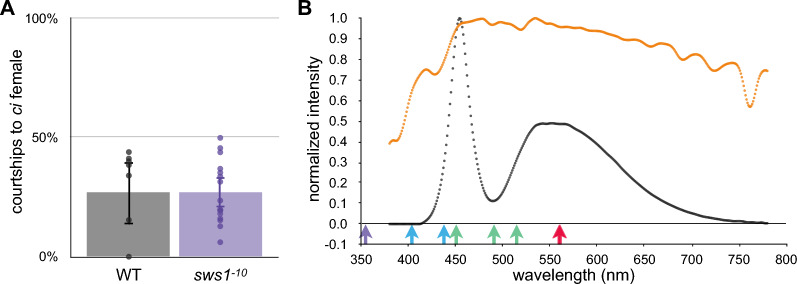
Mate choice of the *sws1* mutant. **a** Body-color preference of Actb–SLα:GFP males without (black, *n* = 8) or with (violet, *n* = 16) the *sws1*^*−10*^ mutation. The males were given a choice between females of the Actb–SLα:GFP and *ci* strains, and the ratio of courtship events toward the *ci* females was plotted. Each dot represents one male, and the graph reports the mean value. The error bars are the 95% confidence intervals. Regardless of the presence or absence of SWS1, the males significantly preferred Actb–SLα:GFP females, and the ratios were not statistically different between the WT and the mutant fish (*P* = 0.977, Student’s *t*-test). **b** Normalized spectra of the white LED light used for breeding (black) and the sunlight (orange) measured at every 1 nm using a C-7000 Spectromaster (Sekonic). The colored arrows at the bottom indicate the absorption maxima (λ_max_) of the medaka cone opsins [45]: SWS1 (violet); SWS2a and SWS2b (blue); RH2a, RH2b, and RH2c (green); and LWSa and LWSb (red)

Similarly, in this study, the preference of the Actb–SLα:GFP fish (*n* = 8) could clearly be reproduced (Fig. 1a, left); only 13.7%–39.5% (95% confidence interval [CI]) of the courtship attempts of males were directed to *ci* females. The Actb–SLα:GFP fish carrying the *sws1*^*–10*^ mutation (a 10-base deletion in the *SWS1* gene [24]) (*n* = 16) also preferred Actb–SLα:GFP females (Fig. 1a, right); only 20.4%–33.2% of the courtship events were directed to *ci* females. The means of these preferences (26.8% and 26.6%) were not significantly different between the WT and violet-color-blind fish (*P* = 0.977, Student’s *t*-test).

This result (i.e., absence of a reduction in body-color preference in the *sws1* mutant) was in contrast to the reduced body-color preferences of the *lws* [23] or *sws2* [5] mutants, probably because our breeding and experiments were carried out under UV-free conditions (Fig. 1b). Although the fish may behave differently in the open air or under a light source containing UV light, the role of SWS1-expressing cones in the body-color preference seemed to be negligible under the UV-free condition.

### OMR of the *sws1* and *sws1/sws2*-double mutants under UV light

Taking advantage of the existing *sws1* [24] and *sws2* [5] mutants, we established a *sws1/sws2*-double mutant, anticipating that the potential reduction in UV sensitivity could be detected more clearly by the violet/blue-double-color-blind fish than by the violet-color-blind fish. We crossed a *sws1*^*−10*^ homozygote with a *sws2*^+*1a*+*14b*^ homozygote, which possessed 1-base and 14-base insertions in the tightly linked *SWS2a* and *SWS2b* genes, respectively [5]; raised the offspring (F_1_), which were double-heterozygous for the *sws1*^*−10*^ and *sws2*^+*1a*+*14b*^ mutations (more accurately, triple-heterozygous for the *sws1*^*−10*^, *sws2a*^+*1*^, and *sws2b*^+*14*^ mutations); and intercrossed the F_1_ to obtain F_2_ individuals.

**Fig. 2:**
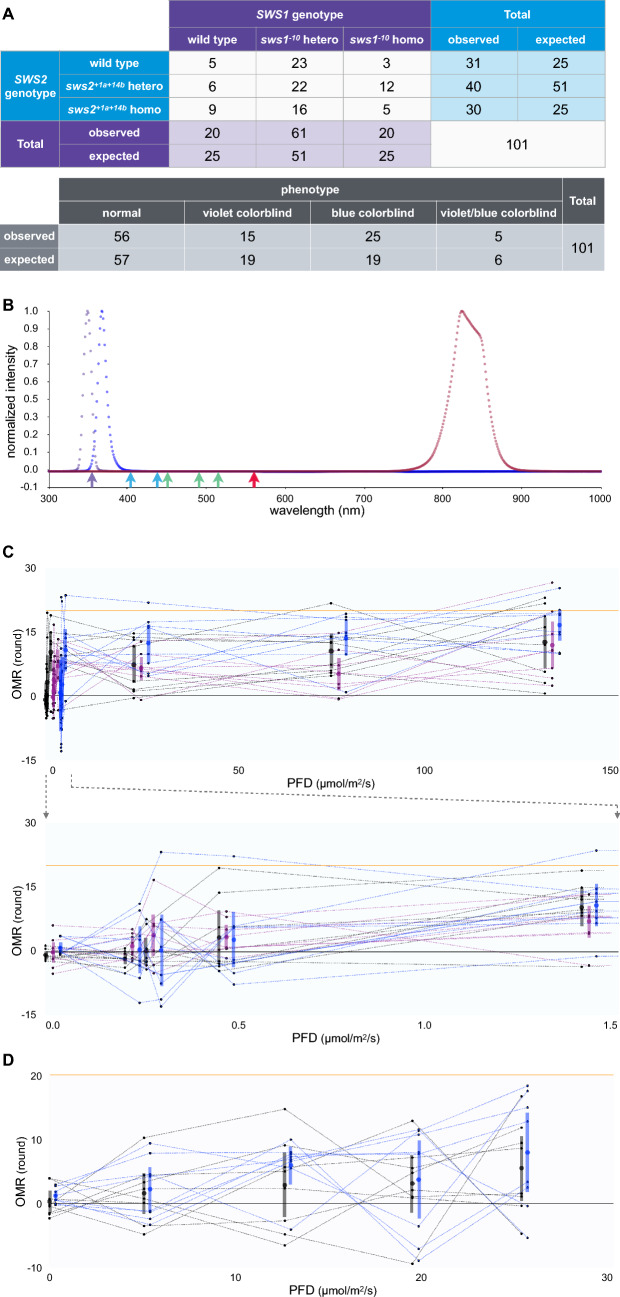
Behavioral UV sensitivity of the *sws1* or *sws1/sws2* double mutants. **a** Establishment of the *sws1/sws2* double mutants. Double heterozygotes for the *sws1*^*−10*^ and *sws2*^+*1a*+*14b*^ mutations were intercrossed, and the offspring were raised under identical conditions until maturation; their genotypes (top) and phenotypes (bottom) are summarized in the tables. No significant difference was detected between the observed and expected ratios. **b** Normalized spectra of the UV light used for the experiments in **c** (blue) and **d** (violet). An IR spectrum of the IR camera for video recording is also shown (dark red). These spectra were measured at every 1 nm using a Sun Spectroradiometer S-2440 instrument (Soma Optics). **c** OMR under UV light (λ = 365 nm). Eight intensities of 0.0, 0.21, 0.27, 0.47, 1.4, 24, 77, and 130 μmol/m^2^/s (as measured by the Sun Spectroradiometer S-2440) were applied. The graphs of the WT (black), *sws1* mutant (purple), and *sws1/sws2* double mutant (blue) (*n* = 8 each) are horizontally shifted for viewing purposes. Each dot represents a result of one fish, and the results of the same fish at different intensities are connected by straight lines. The closed circles and vertical bars indicate the mean and the 95% confidence intervals of the mean, respectively. The horizontal orange line at the OMR of 20 rounds indicates that the fish perfectly followed the rotating stripes (10 rpm × 2 min). **d** OMR under UV light at a shorter wavelength (λ = 350 nm). We compared the WT and the *sws1/sws2* double mutant (*n* = 8 each) at five intensities of 0.0, 5.1, 13, 20, and 25 μmol/m^2^/s. See (**c**) for details

We reared the F_2_ fish in the same tanks *en masse* (the genotypes could not be determined based on their appearance), 101 of which matured fully. Because the *SWS1* and *SWS2a/b* loci are independent [31], the expected phenotypic ratio of WT, violet-color-blind, blue-color-blind, and violet/blue-double color-blind fish was 9:3:3:1, which was indeed observed in the F_2_ generation (*P* = 0.385, chi-square test; Fig. 2a). Therefore, not only the *sws1* or *sws2* mutants [5, 23], but also the *sws1/sws2*-double mutant, were as viable as their WT litter mates with normal color vision, at least in our breeding conditions.

Subsequently, we investigated the OMR of the WT, *sws1*-mutant, and *sws1/sws2*-double-mutant individuals under UV light (λ = 350 or 365 nm). The spectra of the UV light for experiments are shown in Fig. 2b, together with that of the IR light for videorecording. We verified that IR light alone did not induce the OMR (see the graphs at the photon flux density [PFD] of 0.0 μmol/m^2^/s in Fig. 2c, d).

At 365 nm, we examined eight PFD values, i.e., 0.00, 0.21, 0.27, 0.47, 1.44, 23.7, 76.7, and 134 μmol/m^2^/s (Fig. 2c). Importantly, all three strains (WT, the *sws1* mutant, and the *sws1/sws2*-double mutant; *n* = 8 each) exhibited the OMR at a PFD ≥ 1.44 μmol/m^2^/s (note that the 95% CI did not include zero in all three strains). This result clearly demonstrated that medaka could perceive and behaviorally respond to UV light, even without SWS1 and SWS2. The OMR observed at a lower PFD was weaker (e.g., the mean of less than four rounds, and the lower limit of the 95% CI being close to zero) in all strains. Statistically, a two-way repeated-measures analysis of variance (ANOVA) detected a significant difference among the eight UV conditions (F_(4.015, 80.307)_ = 18.723, *P* < 0.001, η_p_^2^ = 0.484), but not among the three strains (F_(2, 20)_ = 1.516, *P* = 0.244, η_p_^2^ = 0.132). No interaction was detected between the UV condition and the strain (F_(8.031, 80.307)_ = 1.118, *P* = 0.360, η_p_^2^ = 0.101). That is, contrary to our expectations, the presence or absence of SWS1 and SWS2 did not significantly affect the behavioral UV sensitivity.

At 350 nm, we examined the OMR of the WT and the *sws1/sws2*-double-mutant individuals (*n* = 8 each) at five PFD values of 0.00, 5.08, 12.6, 19.5, and 25.4 μmol/m^2^/s (Fig. 2d). We occasionally observed that the test fish uncomfortably twisted their body when the UV light was turned on. This could explain why a reverse OMR (swimming against the rotating stripes) was often observed at high UV intensities (e.g., 19.5 μmol/m^2^/s); i.e., the fish might try to escape from (rather than stay still within) the UV-light-dominated environment. Although the 95% CIs indicated that the OMR was positive at 25.4 μmol/m^2^/s in both strains (note that a few fish exhibited a nearly perfect OMR; i.e., 20 rounds), a two-way repeated-measures ANOVA did not support a significant difference among the five UV conditions (F_(2.579, 30.946)_ = 2.065, *P* = 0.141, η_p_^2^ = 0.133) or between the strains (F_(1, 12)_ = 0.607, *P* = 0.451, η_p_^2^ = 0.048). Their interaction was not significant (F_(2.579, 30.946)_ = 0.060, *P* = 0.990, η_p_^2^ = 0.005).

It should be noted that all test fish were light-adapted prior to, and the rods were dysfunctional during, the OMR tests [2]; i.e., the UV light must be perceived via RH2 and/or LWS, rather than rhodopsin (RH1), in the *sws1/sws2*-double mutant, although other non-canonical photoreceptors could also be involved (further discussed below).

### Establishment of a new OMR-testing device

In the experiments described above (Fig. 2c, d), although we paid great attention to avoiding any contamination of fluorescent light excited by UV (e.g., wrapping all the devices in aluminum foil, using vertical stripes made of strips of aluminum foil pasted on an Indian-ink-painted plastic paper, wearing gloves to avoid leaving fingerprints), there might have been some human-undetectable fluorescence that the medaka perceived and responded to. Therefore, we further investigated the behavioral photosensitivity of the *sws1/sws2*-double mutant in UV-free conditions.

Equiluminance (isoluminance) is an equally luminant condition between different colors, in which the recognition of differences becomes the most difficult. Hence, when the rotating stripes consisted of equiluminant colors (e.g., equally luminant green and red), the OMR should be minimized compared with that elicited by non-equiluminant colors.

**Fig. 3:**
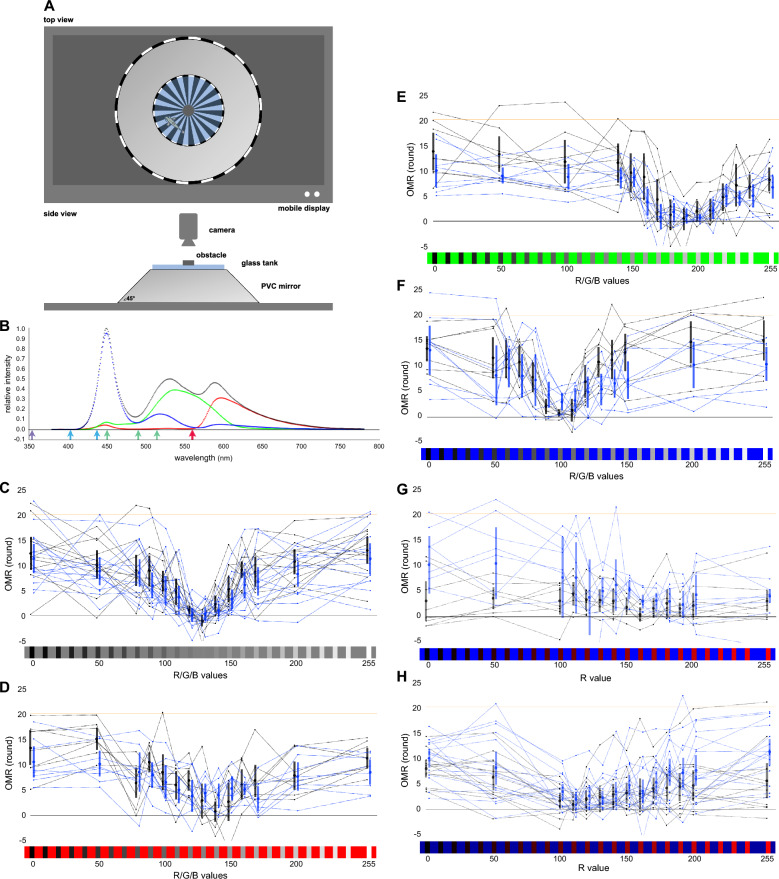
Equiluminant conditions defined by the OMR. **a** Experimental setup. Spinning sunray-shaped stripes projected onto the display at the bottom were reflected horizontally into rotating vertical stripes by a polyvinyl-chloride mirror. **b** Normalized spectra from the display (MB16AP; Asus) used in (**c**–**h**). White (RGB values of 255/255/255), red (255/0/0), green (0/255/0), and blue (0/0/255) light were measured at every 1 nm using a Spectromaster C-7000 (Sekonic) and are shown in gray, red, green, and blue, respectively. **c** OMR in gray–gray stripes. One gray was fixed at 128/128/128, and the other was set at either 0/0/0, 50/50/50, 80/80/80, 90/90/90, 100/100/100, 110/110/110, 120/120/120, 130/130/130, 140/140/140, 150/150/150, 160/160/160, 170/170/170, 200/200/200, or 255/255/255. See Fig. 2c for details. Some data points are beyond the graph area. Black, wild-type fish; blue, *sws1/sws2* double mutant. **d** OMR in red–gray stripes. Red was fixed at 255/0/0, and gray was either 0/0/0, 50/50/50, 80/80/80, 90/90/90, 100/100/100, 110/110/110, 120/120/120, 130/130/130, 140/140/140, 150/150/150, 160/160/160, 170/170/170, 200/200/200, or 255/255/255. **e** OMR in green–gray stripes. Green was fixed at 0/255/0, and gray was either 0/0/0, 50/50/50, 100/100/100, 140/140/140, 150/150/150, 160/160/160, 170/170/170, 180/180/180, 190/190/190, 200/200/200, 210/210/210, 220/220/220, 230/230/230, 240/240/240, or 255/255/255. **f** OMR in blue–gray stripes. Blue was fixed at 0/0/255, and gray was either 0/0/0, 50/50/50, 60/60/60, 70/70/70, 80/80/80, 90/90/90, 100/100/100, 110/110/110, 120/120/120, 130/130/130, 140/140/140, 150/150/150, 200/200/200, or 255/255/255. **g** OMR in blue–red stripes. Blue was fixed at 0/0/255, and red was either 0/0/0, 50/0/0, 100/0/0, 110/0/0, 120/0/0, 130/0/0, 140/0/0, 150/0/0, 160/0/0, 170/0/0, 180/0/0, 190/0/0, 200/0/0, or 255/0/0. **h** OMR in dark-blue–red stripes. Dark blue was fixed at 0/0/160, and red was either 0/0/0, 50/0/0, 80/0/0, 90/0/0, 100/0/0, 110/0/0, 120/0/0, 130/0/0, 140/0/0, 150/0/0, 160/0/0, 170/0/0, 180/0/0, 190/0/0, 200/0/0, or 255/0/0

To test this hypothesis, we established the new experimental system shown in Fig. 3a. Briefly, the test fish were placed in a cylindrical tank surrounded by a truncated-cone-shaped mirror, and spinning fan-shaped stripes on a display placed below the tank were horizontally reflected by the mirror, to present rotating vertical stripes to the fish (the OMR could not be induced without the mirror, unlike that observed in zebrafish). The spectra of white, red, green, and blue light from the display are shown in Fig. 3b.

We first tested this system using gray–gray stripes (Fig. 3c), in which one gray was fixed at an RGB value of 128/128/128 and the other gray was set at 14 luminance (from 0/0/0 [black] to 255/255/255 [white]). As expected, the OMR of the WT (*n* = 12) and the *sws1/sws2*-double mutant (*n* = 14) fish was minimized in the case of the 128/128/128–120/120/120 or 128/128/128–130/130/130 stripes, a condition in which the stripes became the most difficult to be recognized. The variance of the data was significantly different among the conditions (χ^2^ (90) = 145.125, *P* < 0.001, Mauchly’s test of sphericity); however, a two-way repeated-measures ANOVA detected significant differences among the stripe conditions (F_(7.425, 178.190)_ = 28.547, *P* < 0.001, η_p_^2^ = 0.543), but not between the strains (F_(1, 24)_ = 2.002, *P* = 0.170, η_p_^2^ = 0.077). No significant interaction was detected in between (F_(7.425, 178.190)_ = 0.554, *P* = 0.802, η_p_^2^ = 0.023).

### Equiluminant conditions for the *sws1/sws2* double mutants

Next, we changed the fixed gray color (128/128/128) to a red (255/0/0), green (0/255/0), or blue (0/0/255) color and tested the OMR of the WT and *sws1/sws2*-double-mutant fish (*n* = 8 each). We expected that, if the sensitivity to blue light was decreased in the double mutant, the blue would be equiluminant to, and therefore the OMR would be minimized in the presence of, the darker gray color in the double mutant versus the WT fish.

In the presence of red–gray stripes (Fig. 3d), the OMR was apparently positive in the extreme (i.e., red–black or red–white) conditions, thus demonstrating that these stripes were clearly visible to the WT and double-mutant fish. However, the graphs adopted a broad U shape and the condition at which the OMR was minimized was difficult to identify, particularly for the double mutant, which might have caused the significant interaction observed between the stripe conditions and the strains (F_(13, 182)_ = 25.785, *P* = 0.003, η_p_^2^ = 0.156). A two-way repeated-measures ANOVA detected significant differences among the stripe conditions (F_(13, 182)_ = 15.181, *P* < 0.001, η_p_^2^ = 0.520), but not between the strains (F_(1, 14)_ = 0.606, *P* = 0.449, η_p_^2^ = 0.042).

In the presence of green–gray stripes (Fig. 3e), the OMR was minimized at a brighter gray color (190/190/190 or 200/200/200) compared with the red–gray stripes (at 140/140/140 for the WT fish). Therefore, medaka should detect the green light (0/255/0) to a greater extent than it does the red light (255/0/0), as humans do. The graphs appeared similar between the WT fish and double mutants, and no interaction was detected between the stripe conditions and strains (F_(13, 182)_ = 0.975, *P* = 0.477, η_p_^2^ = 0.065). A two-way repeated-measures ANOVA detected significant differences among the stripe conditions (F_(13, 182)_ = 16.512, *P* < 0.001, η_p_^2^ = 0.541), but not between the strains (F_(1, 14)_ = 3.007, *P* = 0.105, η_p_^2^ = 0.177).

In the presence of blue–gray stripes (Fig. 3f), the OMR was minimized at a darker gray color (100/100/100 or 110/110/110) compared with the green–gray or red–gray stripes, demonstrating that medaka detect the blue light to a lesser extent than the red or green light, similar to humans. The graph of the double mutants appeared to be flatter than that of the WT fish (as in the red–gray stripes; Fig. 3d), and a significant interaction was detected between the stripe conditions and strains (F_(13, 182)_ = 2.442, *P* = 0.005, η_p_^2^ = 0.149). The dark shift of the equiluminant condition that was expected in the double mutant seemed not to occur. In fact, significant differences were detected among the stripe conditions (F_(13, 182)_ = 17.353, *P* < 0.001, η_p_^2^ = 0.553), but not between the strains (F_(1, 14)_ = 1.278, *P* = 0.277, η_p_^2^ = 0.084). Thus, the *sws1/sws2* double-mutant fish seemed to sense the blue as luminant as the WT fish did.

### Equiluminant red and blue for the *sws1/sws2* double mutants

The “gray” color, however, consists of red, green, and “blue” light (Fig. 3b). Therefore, the reduction in blue-light sensitivity would also reduce the sensitivity to gray, which could explain why the dark shift could not be detected in the presence of blue–gray stripes (Fig. 3f). Therefore, supposing that the lack of SWS1 and SWS2 should least affect the sensitivity to red light, we repeated the OMR test by replacing the variable gray color (0/0/0–255/255/255) with variable red color (0/0/0–255/0/0).

First, we fixed the blue color at 0/0/255 (Fig. 3g); however, the fish (*n* = 8 each for the WT and the double mutant) were not “cooperative” with the test (e.g., one mutant exhibited the reverse OMR in 10 of 14 stripe conditions), and a two-way repeated-measures ANOVA detected no significant difference among the stripe conditions (F_(2.997, 41.958)_ = 2.550, *P* = 0.069, η_p_^2^ = 0.154). It also seemed that the blue (0/0/255), which was equiluminant to the gray of 100/100/100 (Fig. 3f), was too luminant to induce the OMR with the brightest red (255/0/0), which was equiluminant to the gray of 140/140/140 (Fig. 3d), sufficiently.

Therefore, we darkened the fixed blue (from 0/0/255 to 0/0/160) and repeated the OMR test using different fish (*n* = 13 or 16 for the WT or the double mutant, respectively) (Fig. 3h). The graphs adopted a broad, but flat, U shape (compared with those depicted in Fig. 3c–f) in the two strains, likely reflecting a milder luminance shift in the varying red (i.e., from 0/0/0 to 255/0/0) than in the varying gray (i.e., from 0/0/0 to 255/255/255). The OMR seemed to be minimized at 110/0/0 in the two strains. A two-way repeated-measures ANOVA detected a significant difference among the stripe conditions (F_(4.725, 127.572)_ = 12.534, *P* < 0.001, η_p_^2^ = 0.317), but not between the strains (F_(1, 27)_ = 2.934, *P* = 0.098, η_p_^2^ = 0.098). No interaction was detected between the stripe condition and the strain (F_(4.725, 127.572)_ = 1.211, *P* = 0.308, η_p_^2^ = 0.043).

Taken together, neither the OMR elicited under UV (Fig. 2) nor RGB (Fig. 3) light supported the reduced behavioral UV or blue-light sensitivity in the *sws1/sws2*-double-mutant medaka.

### Establishment of the *sws1/sws2/lws* triple mutant medaka

**Fig. 4:**
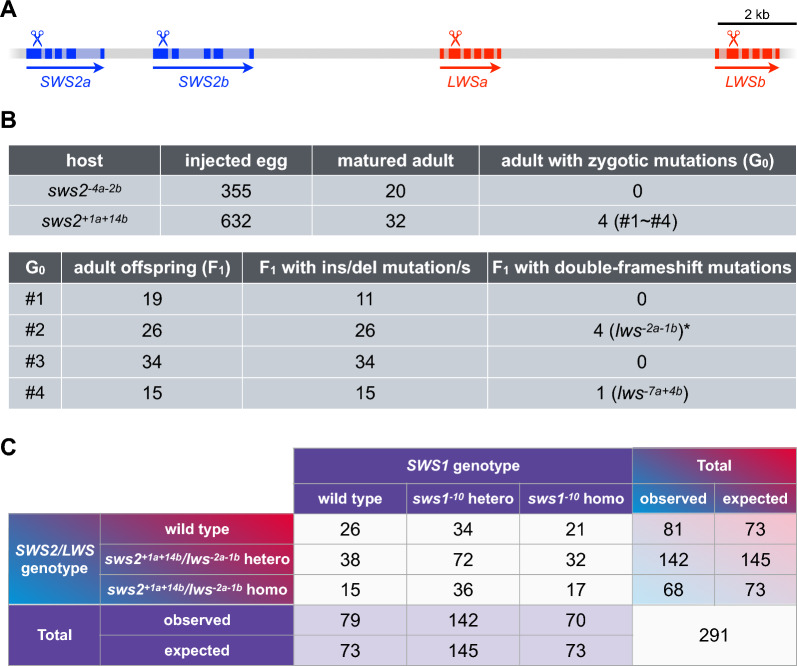
Establishment of the *sws1/sws2/lws* triple mutant (i.e., the RH2 monochromat). **a** Genomic structure of the *SWS2* (blue) and *LWS* (red) loci. Each locus consists of two paralogous genes (*a* and *b*). The arrows and colored boxes indicate the directions of transcription and the translated regions, respectively. The scissors indicate the approximate positions of the target sequences for CRISPR/Cas9 [2, 4, 5]. **b** Induction of the *lws* mutations in the *sws2* mutant. Top: Production of mosaic mutants (G_0_) by microinjection. We obtained four G_0_ adults that had the ins/del mutations in the caudal fin. Bottom: Transmission of mutations from the G_0_ fish to their offspring (F_1_). The asterisk indicates that all four F_1_ fish inherited identical mutations; 2- or 1-base deletions in the *LWSa* and *LWSb* genes, respectively. **c** Production of the triple mutant by crossing. The *sws2*^+*1a*+*14b*^*/lws*^*−2a−1b*^ double mutant was crossed with the *sws1*^*−10*^ mutant, and their offspring (*sws1*^*−10*^*/sws2*^+*1a*+*14b*^*/lws*^*−2a−1b*^ triple heterozygotes) were intercrossed. The genotypes of the mature offspring (F_2_) are summarized in the table

To characterize further the UV perception via the green and/or red opsins, we established and analyzed a strain that possessed frameshift mutations in the *SWS1*, *SWS2a*, *SWS2b*, *LWSa*, and *LWSb* genes; i.e., the *sws1/sws2/lws* triple mutant. Because the *SWS2a/b* and *LWSa/b* loci are tightly linked on a chromosome [31], it was nearly impossible to establish the *sws2/lws* double mutant by crossing the existing *sws2* [5] and *lws* [2] mutants. Therefore, we newly introduced *lws* mutations in the *sws2* mutant (Fig. 4a, b). A total of four adult fish (G_0_) possessed and passed the ins/del mutations in the *LWSa/b* genes to their offspring (F_1_), five of which carried the double-frameshift mutations, *lws*^*−2a−1b*^ or *lws*^*−7a*+*4b*^. Although the *lws*^*−7a*+*4b*^ mutation was unfortunately lost during later crossings, we were able to establish a line that was homozygous for the *sws2*^+*1a*+*14b*^ and *lws*^*−2a−1b*^ mutations, i.e., the *sws2/lws* double mutant.

This double mutant was then crossed with the *sws1*^*−10*^ mutant, and the *sws1/sws2/lws* triple heterozygotes (more precisely, *sws1*^*−10*^*/sws2a*^+*1*^*/sws2b*^+*14*^*/lwsa*^*−2*^*/lwsb*^*−1*^ quintuple heterozygotes) were intercrossed to obtain the *sws1/sws2/lws* triple mutant at the probability of 1/16 (the *SWS1* and *SWS2/LWS* loci are independent [31]). We raised a total of 291 fish into the adult stage; their genotypes are summarized in Fig. 4c. The WT:hetero:homo ratio in the *SWS1* or *SWS2/LWS* loci was not significantly different from 1:2:1 (*P* = 0.696 and 0.514, respectively; chi-square test), demonstrating that not only the *sws1* [24], *sws2* [5], *lws* [2], and *sws1/sws2* double (Fig. 2a) mutants, but also the *sws2/lws* double and *sws1/sws2/lws* triple mutants, were fully viable in our breeding conditions. All color-blind mutants were indistinguishable based on appearance.

### Expression of the *cone-opsin* genes in the *sws1/sws2/lws* triple mutant

**Fig. 5:**
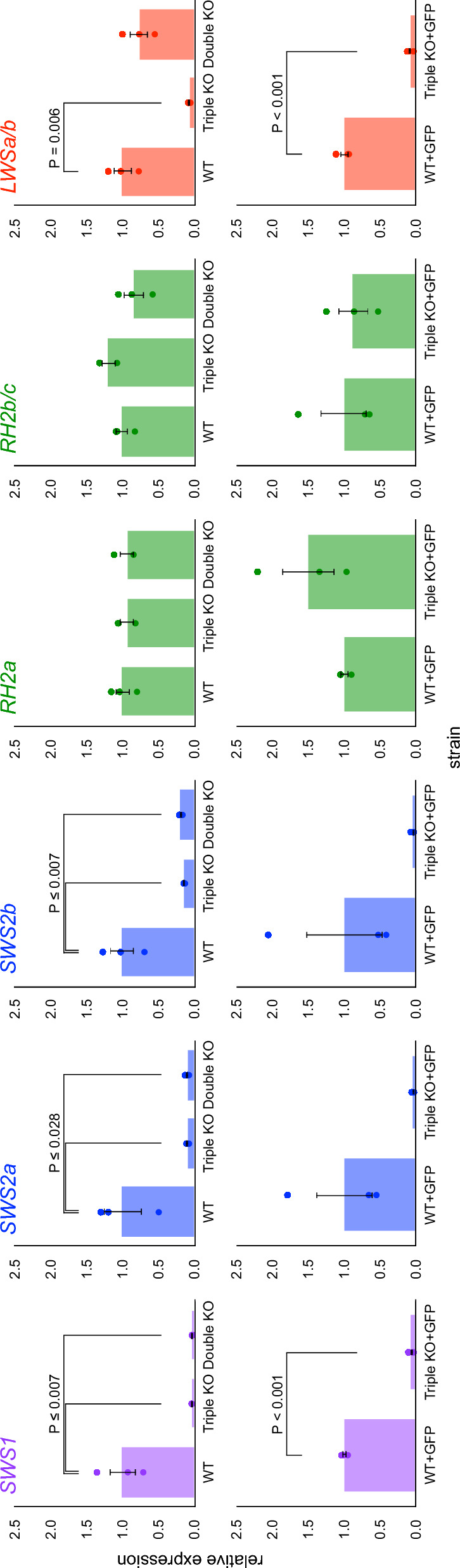
Expression of the *cone-opsin* genes in the *sws1/sws2/lws* triple mutant. The expression levels of the *SWS1*, *SWS2a*, *SWS2b*, *RH2a*, *RH2b/c*, and *LWSa/b* genes in the mutant relative to those in the WT were quantified by the ΔΔCt method using the *Actb* gene as a reference. The *RH2a* and *RH2b* genes and the *LWSa* and *LWSb* genes were indistinguishably amplified because of similar nucleotide sequences. Top: Comparison on the *ci* background (the *sws1/sws2* double mutant was included). Bottom: Comparison on the Actb–SLα:GFP background. Each dot represents one individual, and the graph shows the mean and the standard error. Significant differences are indicated by the *P* value (top: one-way ANOVA and post-hoc Dunnett’s test; bottom: Student’s two-tailed *t-*test)

We considered that the color-blind mutations might increase the expression of the remaining cone opsins to compensate for the decreased repertoire (e.g., the *sws1/sws2/lws* triple mutant might express the remaining RH2 more strongly compared with the WT fish). We previously found that the *cone-opsin* genes were differently transcribed between *ci* and Actb–SLα:GFP, possibly because of the ectopic expression of *Renilla* GFP [5]. Therefore, we compared gene expression independently on the *ci* or Actb–SLα:GFP background using real-time reverse transcription polymerase chain reaction (RT-PCR) (Fig. 5).

On the *ci* background, we compared the WT (*n* = 3), the *sws1/sws2/lws* triple mutant (*n* = 2), and the *sws1/sws2* double mutant (*n* = 3) fish. An apparent reduction caused by nonsense-mediated mRNA decay (NMD) could be detected for the *SWS1*, *SWS2a*, *SWS2b*, and *LWSa/b* genes (*LWSa* and *LWSb* are 98.8% identical, and we analyzed them without discrimination) in the triple mutant and for the *SWS1*, *SWS2a*, and *SWS2b* genes in the double mutant (*P* ≤ 0.018, one-way ANOVA and post-hoc Dunnett’s test). By contrast, the expression of *RH2a* and *RH2b/c* (*RH2b* and *RH2c* are 95.8% identical, and we analyzed them without discrimination) was equivalent among the three strains (*P* = 0.891 or 0.220, respectively; one-way ANOVA).

On the Actb–SLα:GFP background, we compared the WT and the triple-mutant fish (*n* = 3 each). An apparent reduction triggered by NMD could be verified for the *SWS1* and *LWSa/b* genes (*P* < 0.001, Student’s two-tailed *t*-test). However, the reduction in the *SWS2a* or *SWS2b* genes was not statistically significant (*P* = 0.073 or 0.146, respectively), likely because one WT individual expressed *SWS2*s (and also *RH2b/c*) very strongly, for unknown reasons. For *RH2a* and *RH2b/c*, significant differences were not detected between the WT and the triple-mutant fish (*P* = 0.243 or 0.769, respectively).

### Spectral sensitivity of the *sws1/sws2/lws* triple mutant

**Fig. 6:**
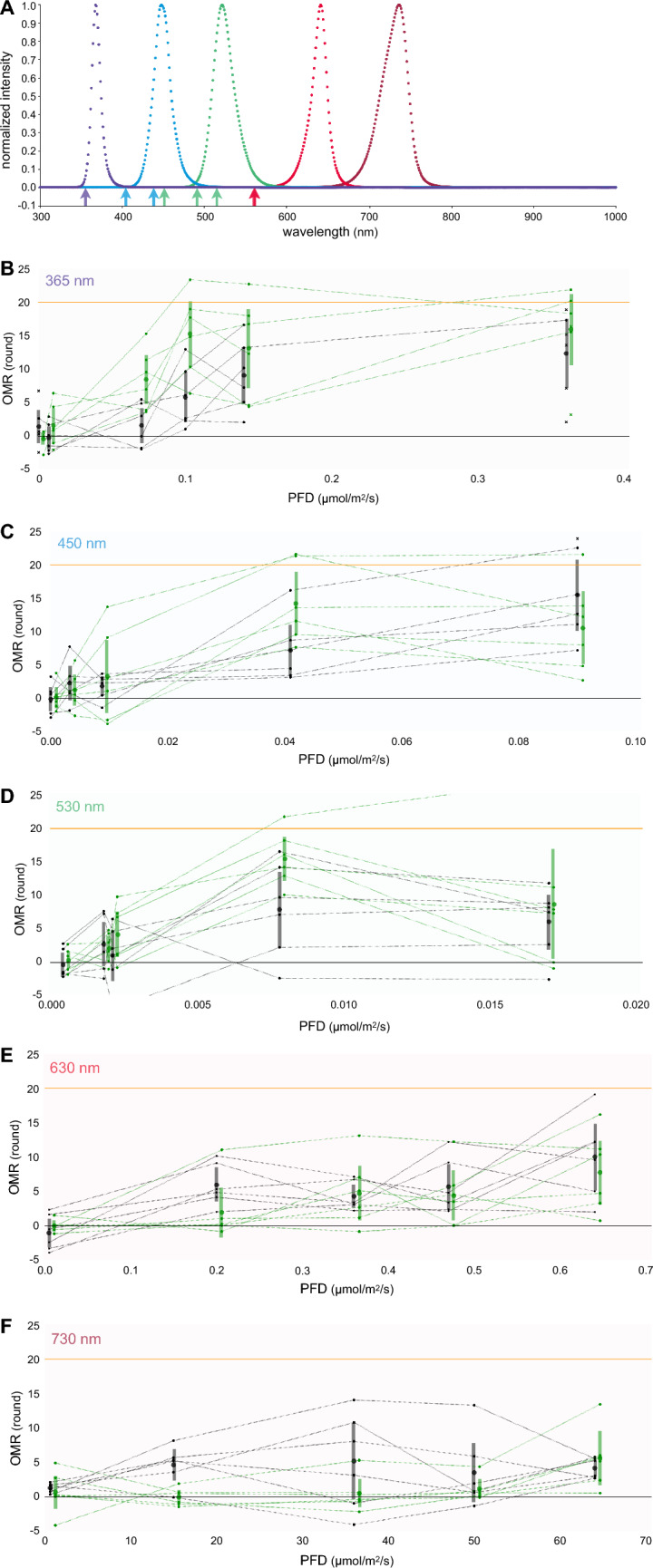
Spectral sensitivity of the *sws1/sws2/lws* triple mutant. **a** Spectra of the LED light used for the OMR test in (**b**–**f**). The peak wavelengths should be 365, 450, 530, 630, and 730 nm according to the manufacturer; however, the data measured by the Sun Spectroradiometer S-2440 instrument (Soma Optics) showed that they were 367, 447, 521, 641, and 736 nm. **b** OMR under UV light (λ = 365 nm). The wild-type (*n* = 6; black) and triple-mutant (*n* = 6; green) fish were tested under seven PFD values of 0.0, 6.8 × 10^–3^, 7.0 × 10^–2^, 1.0 × 10^–1^, 1.4 × 10^–1^, 3.6 × 10^–1^, and 8.4 μmol/m^2^/s (as measured by the QTM-101 quantameter; Monotech). See Fig. 2c for details. Data of the replaced fish (see Results) were shown by cross marks (instead of dots). The results obtained at 8.4 μmol/m^2^/s (9.1–16.4 and 14.0–19.7 rounds [95% confident intervals] in the WT and triple mutant, respectively) were omitted from the graph. **c** OMR under blue light (λ = 450 nm) tested at 8.8 × 10^–5^, 3.3 × 10^–3^, 8.8 × 10^–3^, 4.1 × 10^–2^, and 9.0 × 10^–2^ μmol/m^2^/s. **d** OMR under green light (λ = 530 nm) tested at 4.2 × 10^–4^, 1.8 × 10^–3^, 2.1 × 10^–3^, 7.8 × 10^–3^, or 1.7 × 10^–2^ μmol/m^2^/s. **e** OMR under red light (λ = 630 nm) tested at 5.3 × 10^–3^, 2.0 × 10^–1^, 3.6 × 10^–1^, 4.7 × 10^–1^, or 6.4 × 10^–1^ μmol/m^2^/s. **f** OMR under near-IR light (λ = 730 nm) tested at 6.7 × 10^–1^, 1.5 × 10^+1^, 3.6 × 10^+1^, 5.0 × 10^+1^, or 6.4 × 10^+1^ μmol/m^2^/s

Lastly, we examined the spectral photosensitivity of the triple mutant via the OMR test under monochromatic light at five wavelengths (λ = 365, 450, 530, 630, or 730 nm; Fig. 6), the spectra of which are presented in Fig. 6a. We set five or six luminance conditions for each wavelength and used six fish per strain per condition; however, some fish died and needed to be replaced during the experiments, particularly at 365 nm.

At 530 or 630 nm (Fig. 6d, e), which were values at which no fish died during the experiments, a two-way repeated-measures ANOVA detected significant differences in the OMR among the luminance conditions (F_(4, 40)_ = 14.443 or 10.164, *P* < 0.001, η_p_^2^ = 0.591 or 0.504, respectively), but not between the strains (F_(1, 10)_ = 1.858 or 0.673, *P* = 0.203 or 0.431, η_p_^2^ = 0.157 or 0.063, respectively). No interaction was detected between the luminance and the strain (F_(4, 40)_ = 1.590 or 0.933, *P* = 0.196 or 0.455, η_p_^2^ = 0.137 or 0.085, respectively).

At 450 nm or 730 nm (Fig. 6c, f), one WT or one mutant fish, respectively, died during the experiments, and we compensated the lacking data (namely, at 0.09 or 50 μmol/m^2^/s, respectively) using a different fish. Supposing that the data were obtained from the original fish, we performed a two-way repeated-measures ANOVA. At 450 nm, the OMR was significantly different among the luminance conditions (F_(4, 40)_ = 24.194, *P* < 0.001, η_p_^2^ = 0.708), but not between the strains (F_(1, 10)_ = 0.102, *P* = 0.756, η_p_^2^ = 0.010), although the interaction was significant (F_(4, 40)_ = 3.361, *P* = 0.018, η_p_^2^ = 0.252). The results of a two-way repeated-measures ANOVA excluding the dead fish (i.e., *n* = 5 or 6 for the WT or the triple-mutant fish, respectively) were basically the same (i.e., the difference was significant among the luminance but not between the strains), with the exception that the interaction became not significant (F_(4, 36)_ = 2.280, *P* = 0.080, η_p_^2^ = 0.202).

At 730 nm, the OMR was not significantly different among the luminance values (F_(4, 40)_ = 2.499, *P* = 0.058, η_p_^2^ = 0.200); i.e., although the OMR seemed to be positive at 64 μmol/m^2^/s in both strains (i.e., the 95% CIs did not include zero), it was not statistically different from that observed at 0.67 μmol/m^2^/s. The wavelength of 730 nm is that at which the *lws* mutant slightly showed a reduced OMR in our previous experiments [2, 3]. In fact, the OMR seemed to be reduced in the triple mutant at weaker intensities (e.g., 15 μmol/m^2^/s), but the overall difference between the strains was not significant according to a two-way repeated-measures ANOVA (F_(1, 10)_ = 2.800, *P* = 0.125, η_p_^2^ = 0.219).

At 365 nm, we had to use 11 WT and 10 triple-mutant fish to complete the data (*n* = 6 each at six luminant conditions). The cause of this higher mortality despite the much weaker UV intensities (0.0–8.4 μmol/m^2^/s) compared with those reported in Fig. 2c (0.00–134 μmol/m^2^/s) is unknown; however, the differences in the experimenter (fish handling, schedule for the OMR tests [the number of experiments per fish], etc.), fish condition/age, and/or season (room temperature) should be considerable. The data could not be analyzed using two-way repeated-measures ANOVA; therefore, we adopted the ordinary two-way ANOVA (although some fish were repeatedly measured). The OMR was significantly different among the conditions (F_(5, 60)_ = 16.661, *P* < 0.001, η_p_^2^ = 0.581) and between the strains (F_(1, 60)_ = 11.977, *P* = 0.001, η_p_^2^ = 0.166). Namely, the OMR was significantly “increased in the triple mutant” at 0.07 and 0.1 μmol/m^2^/s (*P* = 0.018 and 0.001, respectively; multiple comparisons with the Bonferroni correction). No significant interaction was detected between the luminance and the strain (F_(5, 60)_ = 1.929, *P* = 0.103, η_p_^2^ = 0.138).

Thus, medaka can fully perceive and behaviorally respond to UV light using RH2 alone, although the involvement of other non-canonical photoreceptors in UV perception could not be excluded (further discussed below). However, this observation should not be surprising because all cone opsins absorb UV light, which is reflected as a secondary peak in the absorption spectrum, i.e., the β band [7], although its absorption was shown to be not greater than about 20% relative to that of the α band in goldfish (see [32]).

## Discussion

Despite our attempts to demonstrate the potential decrease in UV- or blue-light sensitivity in medaka lacking SWS1 and SWS2, none of the results presented here (Figs. 2c, d, 3f–h, 6b, c) supported this assumption. In fact, the UV sensitivity might even be “increased” in the *sws1/sws2/lws* triple mutant (Fig. 6b). The present study should provide an important premise considering the function and evolution of cone opsins in animals; i.e., the presence or absence of a certain type of cone opsin does not necessarily affect the photosensitive behaviors of animals, even at wavelengths close to the λ_max_.

### The OMR as an index of behavioral photosensitivity

The photosensitivity of animals could be measured using various methods (see Introduction), among which, we adopted the OMR in this and previous studies [2–6]. The rationale was simple: when an animal does not follow the rotating stripes, it should be insensitive to the light irradiated from or reflected by the stripes. However, a more careful interpretation of the data seemed to be required.

To elicit the OMR under monochromatic light (i.e., in the condition in which all items exhibit an identical hue), animals must recognize the monochromatic stripes as a difference in luminance (brightness). In primates, the luminance is detected via the medium-wavelength-sensitive (MWS) and LWS opsins (MWS is evolutionary paralogous to LWS); moreover, the contribution of SWS, which is evolutionarily orthologous to SWS1, is restrictive [32, 33].

Our present and previous results of (1) a reduced OMR under red light in the *lws* mutant [2–4] and (2) an OMR in the RH2 monochromat (the *sws1/sws2/lws* triple mutant; Fig. 6b–f) demonstrated that the OMR in medaka depends on both RH2 and LWS. Alternatively, it could be considered that, rather than LWS and RH2, LWS and other non-canonical visual pigments, such as melanopsin, are responsible for the OMR, because there is a growing body of evidence showing their expression in various retinal cells [34, 35] and their actual contribution to vision [36, 37]. In either case, SWS1 and SWS2 should play only a negligible role in the OMR at the present speed (i.e., 10 rpm), considering that the OMR of the *sws1*, *sws2*, or *sws1/sws2* double mutants was not reduced at any wavelength tested in this and previous studies ([5]; Figs. 2c, d, 6b–f).

More than a quarter of a century ago, a similar conclusion had been reached by analyzing the OMR of other fish species. Schaerer and Neumeyer [39] showed that the luminous efficiency function of goldfish (and zebrafish [40]) had a single maximum at the λ_max_ of LWS, and therefore suggested that the LWS-expressing cones were predominantly involved in the OMR; i.e., according to those authors, the motion vision was “color-blind”. A similar result was reported in cichlid [41], whereas not only LWS, but also RH2, seemed to be involved in larval zebrafish [42] and two-spotted goby [43], such as medaka. Thus, SWS1 and SWS2 would commonly be dispensable for the OMR in various fish species. Whether this is a character that is restricted to the OMR or is widely applicable to motion detection (as suggested by Schaerer and Neumeyer [38]) or luminance detection (as known in SWS of primates) warrants further investigation using methods other than the OMR test.

It should be noted that the results described above (ours and those of other researchers) only suggest the negligible role of SWS1 and SWS2 “in relation to that of RH2 and LWS”; i.e., SWS1 and SWS2 might make a significant contribution to the OMR in the absence of RH2 and LWS, and medaka that lack RH2 and LWS (the *rh2/lws* double mutant) might not necessarily be OMR negative. To check this issue, we are currently knocking out three paralogs of the *RH2* gene (*RH2a*, *RH2b*, and *RH2c*); however, the *rh2* mutant seems to be less viable than its WT littermates (our unpublished observation), unlike that observed for the *sws1*, *sws2*, and *lws* mutants [2, 5, 23]. This complicates the interpretation of the data, because even if the *rh2* or *rh2/lws* double mutants exhibit a reduced OMR, this could be attributed to a reduced viability or reduced visual acuity in general, as is known in human SWS monochromats [44].

### Detection of luminance and hue

A much higher light intensity (1.0 × 10^–1^ or 4.1 × 10^–2^ μmol/m^2^/s) was necessary to induce the OMR at 365 or 450 nm, respectively, compared with 530 nm (7.8 × 10^–3^ μmol/m^2^/s; Fig. 6b–d). This result (i.e., luminous efficiency function) consistently supports the negligible roles of SWS1, SWS2b, and SWS2a, and possibly also RH2a (λ_max_ = 356, 405, 439, and 452 nm, respectively [45]), in the OMR. This was in contrast with the result obtained using electroretinography, which showed that the threshold intensity was much lower (i.e., the photosensitivity was much higher) at 380 nm (2.58 × 10^–4^ μmol/m^2^/s) than that observed at 470 or 520 nm (3.90 × 10^–3^ or 8.73 × 10^–4^ μmol/m^2^/s, respectively) in the WT medaka [46]. Therefore, the SWS1-expressing cones should be active during the OMR under UV light, but the action potential was not used for the postreceptoral processes that induce the OMR or, more generally, that detect the luminance (or motion).

About a century ago, Schlieper [47] tested the OMR using colored and gray stripes and found conditions in which the OMR became negative, just as we did in the present study (Fig. 3d–f). His result was initially interpreted as the tested animals being color-blind (see [40]). This interpretation was true in the sense that some fish, such as goldfish or cichlid, exhibited a “color-blind” OMR [38, 40]; i.e., at the equiluminant condition, the alternating colored stripes would virtually disappear for these animals.

The OMR might also be “color-blind” for medaka (and also larval zebrafish and two-spotted goby [41, 42]), in which it relies on both RH2 and LWS, because the OMR of the WT medaka similarly became negative at the equiluminant conditions (Fig. 3d–g). In Fig. 3h, however, the WT medaka consistently exhibited a positive OMR in all conditions, some of which should be equiluminant or near-equiluminant. From this point of view, it was intriguing that the *sws1/sws2* double mutant generally (and statistically significantly) performed better than did the WT fish in the equiluminant conditions; i.e., there was a condition in which the OMR became negative in the WT, but not the mutant fish (e.g., Fig. 3d, f, and h). We interpreted these results as the OMR in medaka not being completely “color-blind”, although the contribution of the hue (RH2–LWS opponency?; [48]) would be relatively subtle compared with that of the luminance.

### Deficiency caused by the lack of SWS1

To date, we have not detected apparent morphological or behavioral defects in medaka lacking SWS1; i.e., the full viability in the laboratory ([24]; Figs. 2a, 4c), the normal cone mosaic in the retina [24], the non-reduced behavioral UV sensitivity (Fig. 2c), and the body-color preference equivalent to that of the WT (Fig. 1a). This is contrasting to the results in larval zebrafish, where acute ablation of the SWS1 cones clearly decreased the OMR and foraging performance [19, 20]. However, these effects in zebrafish larvae were temporal, because the ablated SWS1 cones were rapidly regenerated, which should not be argued the same way with our color-blind medaka that chronically lacks SWS1. The only phenotype we noted was the “increased” OMR in the *sws1/sws2* double mutant in the equiluminant conditions (Fig. 3d, f) and in the *sws1/sws2/lws* triple mutant under UV light (Fig. 6b). Rather than transcriptional upregulation (Fig. 5), the increased UV sensitivity seemed to be achieved by other physiological (e.g., dark adaptation of the RH2-expressing cones) or morphological (e.g., retinomotor movements) mechanisms, which warrant further investigation. The series of color-blind medaka lines would be a useful model to investigate the functional relationships between cone opsin and animal behavior, which should provide an important clue for understanding the evolution of color vision in animals.

## Methods

### Fish

All fish were born and reared in our laboratory, where water was filtrated/circulated at 25 °C and light was provided by white LED for 14 h per day. Fish were given brine shrimps and flake foods five times per day. Sexually mature adults (more than 3 months of age) were used for all experiments.

### Mate choice

A test male was given two choice females in a free-swimming condition (20 × 12 cm with a water level of 5 cm) for 30 min, and the mate preference was manually quantified as a ratio of the male’s approaches. If a male was used in two or more tests, we averaged the ratios and treated this value as a single datum. We judged the preference as being significant if the 95% CI did not contain 50:50.

### Genotyping

A crude extract of genomic DNA from the caudal fin was used as a template for PCR. The primer sequences used here were as follows: f: ACGCCTCTGAACTTTGTCGTTCTTCTG and r: CTTCCAGGGCGCACAGCGTTTG for *SWS1*; f: AACAAGAAGCTTCGATCCCA and r: ATATCTGCAAGCGAAGGAGC for *SWS2a*; f: TTGTTGCTTCTACGGGTTCC and r: TTTGGCTCTAGAGAGGTACAGTCA for *SWS2b*; f: TAAACTGGATTTTGGTCAATCTTGCT and r: CCAACCATCCTCTCAACAGAGC for *LWSa*; and f: CATAGCTGACCTGGGAGAGACG and r: CCAACCATCCTCTCAACAGAGC for *LWSb* (the reverse primers were identical between *LWSa* and *LWSb*). The amplified products were electrophoresed on a 12% polyacrylamide gel, and bands were detected by ethidium-bromide staining and UV irradiation (heteroduplex mobility assay [HMA]).

### OMR test under monochromatic light

The diameters of a cylindrical glass tank and a rotating drum surrounding it were 9 and 15 cm, respectively. A water-filled 2-mL tube wrapped in aluminum foil was placed at the center of the glass tank to prevent shortcut during the OMR. To avoid any fluorescence under UV or blue light, vertical stripes (2-cm wide) were prepared by pasting strips of aluminum foil onto an Indian-ink-painted plastic paper, the device for rotating the drum was covered with pieces of aluminum foil, and we handled all items with gloves to avoid leaving fingerprints on the stripes or device.

Monochromatic light was provided from an LED bulb (EX-365, 450, 530, 630, or 730; Optocode) or a Max-350 xenon lamp (Asahi Spectra) with a bandpass filter of 350 nm. To adjust the intensity, we changed the output or height of the light sources and placed a reflective neutral-density filter in the light path, when necessary. PFD was directly measured using a QTM-101 quantameter (Monotech) or calculated from a spectrum measured by a S-2440 spectroradiometer (Soma Optics). For videorecording, we used an IR camera (ELP-USB100W04H-DL36-J; ELP). Its built-in IR lamps were partly covered with aluminum foil to reduce the intensity; i.e., sufficiently bright for the recording but not for the OMR.

The test fish were light-adapted under ceiling light (Additional file 1: Figure S1) for > 10 min prior to each OMR test, which consisted of a 30-s acclimation and 4 × 30-s rotations in the clockwise, anticlockwise, clockwise, and anticlockwise directions. The speed of stripe rotation was 10 rpm. We quantified the OMR as the swimming distance (rounds) in the direction of stripe rotation during the 120-s rotations. If the fish swam against this direction, the distance was added as a negative value. Therefore, the overall distance should become zero if the fish swam randomly in the dark. The positions of the test fish and the obstacle placed at the center were extracted as x–y coordinates using UMATracker software [49], and were then used for calculating the distance [3].

We calculated the mean distance and its 95% CI per strain per condition and regarded that the OMR was positive when the interval did not contain zero. For a comparison between the WT and the double/triple mutants, we performed a two-way repeated-measures ANOVA or two-way ANOVA depending on the number of fish that died during the experiments (see the Results for details) using SPSS Advanced Statistics software (IBM).

### OMR test for equiluminance

The test fish were placed in the glass tank with the center obstacle (see above). A mobile display (MB16AP; Asus) was laid under the tank, and a polyvinyl-chloride mirror formatted into a conical trapezoid was placed around the tank (see Fig. 3a). The display projected sunray-shaped stripes (36 stripes with a width of 10°) spinning at 10 rpm, and the mirror horizontally reflected the image as rotating vertical stripes. The procedure used for the OMR test was identical; i.e., a 30-s acclimation and 4 × 30-s rotations. The tests were carried out under ordinary fluorescent light from the ceiling whose spectrum was provided as Additional file 1: Figure S1, the behaviors were videorecorded using the C615n webcam (Logicool), and the OMR (swimming distance) was quantified as described above. Because no fish died during these experiments, we applied a two-way repeated-measures ANOVA for statistical comparisons.

### Genome editing

The detailed protocol used for knocking out the *LWS* genes has been described elsewhere [2, 4]. Briefly, the *Cas9* mRNA and guide RNA targeting the 5′–GCGTGTTTGAGGGCTATGTGG–3′ sequence of the paralogous *LWSa* and *LWSb* genes were synthesized and microinjected into embryos at the 1-cell stage. The mutations induced in the caudal fin of the injected fish (G_0_) were detected by an HMA using the appropriate primers, and the mutated G_0_ fish were backcrossed to the host strain. The mutations passed to the offspring (F_1_) were individually sequenced, and the F_1_ fish with identical double-frameshift mutations were intercrossed to obtain homozygotes.

### Real-time RT-PCR

The total RNA was extracted from the eyes of adult fish using ISOGENII (Nippon Gene), contaminated DNA was digested by Doxyribonuclease (RT Grade) for Heat Stop (Nippon Gene), and cDNA was synthesized using ReverTra Ace (Toyobo) and a polyT primer. Real-time RT-PCR was carried out using the innuMIX qPCR DSGreen Standard (Analytik Jena) or the Taq Pro Universal SYBR qPCR Master Mix (Vazyme) on a qTOWER^3^ G touch instrument (Analytik Jena). The thermocycling conditions were as follows: 95 °C for 2 min, followed by 40 cycles of 95 °C for 20 s and 60 °C for 1 min. Primers (Table 1) were designed to sandwich the last intron of each gene and amplify products of about 150 bp. The products were relatively quantified using the ΔΔCt method with the *actin beta* (*Actb*) gene as a reference.

**Table 1:** Primer sequences used for real-time RT-PCR

Gene	Orientation	Sequence (5′ → 3′)
*SWS1*	Forward	TTCTCCAAGAGCTCCTGCGTGTACAA
	Reverse	TTAAGAGGCCGTGGACACCTCCG
*SWS2a*	Forward	TCAAAGGCCTCCACTGTGTACAATCC
	Reverse	CTAAGCTGGTCCGACTTTAGAGACTTC
*SWS2b*	Forward	CCACAGTCTACAACCCCTTCATTTATGTC
	Reverse	TTAGGAAGGGCCGACTTTTGAGACTTC
*RH2a*	Forward	AAAGAGCTCAGCCCTGTTCAATCCTATC
	Reverse	CAAGCAGCAGTAGAGACTTCTGTCTTGC
*RH2b & RH2c*	Forward	AAGAGCTCAGCATTGTACAATGCTGTTATCTA
	Reverse	TTAAGCTGCAGTTGAGACTTCTGTCTTGC
*LWSa & LWSb*	Forward	TTTGCAAAGAGCGCCACAATCTACAACC
	Reverse	TATGCAGGAGCCACAGAGGAGACC
*Actb*	Forward	AGCCCTGGCCCCATCCACCA
	Reverse	GAGGGGCCAGACTCATCGTACTC

### Supplementary Information

**Additional file 1: Figure S1.** A normalized spectrum of the ordinary fluorescent lamps from the ceiling during the OMR tests in Fig. 3.

## Data Availability

The datasets used and/or analyzed during the current study are available from the corresponding author on reasonable request.
