# Supplementary material for: Behavioral photosensitivity of multi-color-blind medaka: enhanced response under ultraviolet light in the absence of short-wavelength-sensitive opsins
Source: BMC Neurosci. 2023 Dec 14;24:67. doi: 10.1186/s12868-023-00835-y (PMC10722765; doi:10.1186/s12868-023-00835-y)
Supplement: Supplementary file 1 — Additional file 1: Figure S1. A normalized spectrum of the ordinary fluorescent lamps from the ceiling during the OMR tests in Fig. 3. [file 12868_2023_835_MOESM1_ESM.pdf]

Supplementary Figure 1

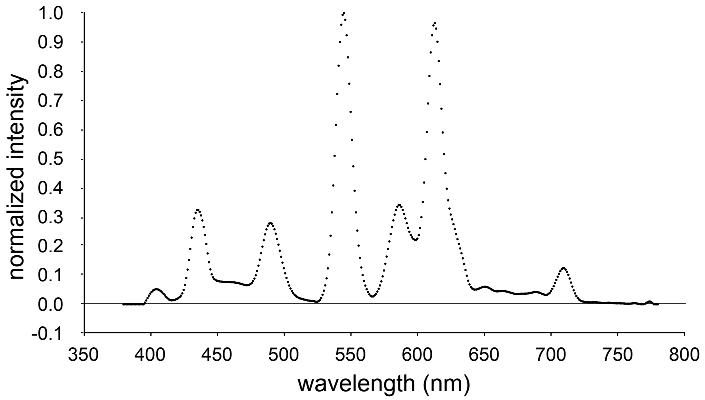

Supplementary Figure 1. A normalized spectrum of the ordinary fluorescent lamps from the ceiling during the OMR tests in Figure 3.
